# A clinical study of kuru patients with long incubation periods at the end of the epidemic in Papua New Guinea

**DOI:** 10.1098/rstb.2008.0068

**Published:** 2008-11-27

**Authors:** John Collinge, Jerome Whitfield, Edward McKintosh, Adam Frosh, Simon Mead, Andrew F. Hill, Sebastian Brandner, Dafydd Thomas, Michael P. Alpers

**Affiliations:** 1 MRC Prion Unit and Department of Neurodegenerative Disease, UCL Institute of Neurology, The National Hospital for Neurology and NeurosurgeryQueen Square, London WC1N 3BG, UK; 2 Papua New Guinea Institute of Medical ResearchPO Box 60, Goroka, EHP 441, Papua New Guinea; 3 Centre for International Health, ABCRC, Shenton Park Campus, Curtin UniversityGPO Box U1987, Perth, WA 6845, Australia

**Keywords:** kuru, prion, spongiform encephalopathy, endocannibalism

## Abstract

Kuru is so far the principal human epidemic prion disease. While its incidence has steadily declined since the cessation of its route of transmission, endocannibalism, in Papua New Guinea in the 1950s, the arrival of variant Creutzfeldt–Jakob disease (vCJD), also thought to be transmitted by dietary prion exposure, has given kuru a new global relevance. We investigated all suspected cases of kuru from July 1996 to June 2004 and identified 11 kuru patients. There were four females and seven males, with an age range of 46–63 years at the onset of disease, in marked contrast to the age and sex distribution when kuru was first investigated 50 years ago. We obtained detailed histories of residence and exposure to mortuary feasts and performed serial neurological examination and genetic studies where possible. All patients were born a significant period before the mortuary practice of transumption ceased and their estimated incubation periods in some cases exceeded 50 years. The principal clinical features of kuru in the studied patients showed the same progressive cerebellar syndrome that had been previously described. Two patients showed marked cognitive impairment well before preterminal stages, in contrast to earlier clinical descriptions. In these patients, the mean clinical duration of 17 months was longer than the overall average in kuru but similar to that previously reported for the same age group, and this may relate to the effects of both patient age and *PRNP* codon 129 genotype. Importantly, no evidence for lymphoreticular colonization with prions, seen uniformly in vCJD, was observed in a patient with kuru at tonsil biopsy.

## 1. Introduction

Kuru is one of the neurodegenerative diseases known as the transmissible spongiform encephalopathies or prion diseases. These diseases affect both animals and humans. The animal prion diseases include scrapie, a naturally occurring disease affecting sheep and goats, that has been recognized for over 200 years and is present in many countries worldwide, and the much more recently recognized bovine spongiform encephalopathy (BSE) among cattle. The human prion diseases have been traditionally classified into Creutzfeldt–Jakob disease (CJD), Gerstmann–Sträussler syndrome, fatal familial insomnia and kuru. These diseases are experimentally transmissible both within and between mammalian species by inoculation with infected tissues and sometimes by dietary exposure. The human prion diseases have three distinct aetiologies: they may be autosomal dominantly inherited conditions; acquired from exposure to environmental prions (via diet or accidental exposure during medical and surgical procedures); or they may arise sporadically (Collinge 2001). Prion diseases are associated with the accumulation of an abnormal, partially protease-resistant isoform of a host-encoded glycoprotein known as prion protein (PrP) in the brain. The disease-related replicating isoform PrP^Sc^ is derived from its normal cellular precursor (PrP^C^) by a post-translational process that involves conformational change and aggregation. According to the ‘protein-only’ hypothesis (Griffith 1967), an abnormal PrP isoform is the principal, and possibly the sole, constituent of the transmissible agent or prion (Prusiner 1982; Collinge & Clarke 2007).

While scrapie has been thought not to infect humans, the large-scale epidemic of BSE in the UK has posed a significant threat to public health and focused much attention on human prion disease. Since 1996, a new human prion disease, variant Creutzfeldt–Jakob disease (vCJD), has been recognized in the UK and experimental studies, involving both molecular and biological strain-typing methods, have confirmed that this is caused by the same prion strain as that causing BSE in cattle (Collinge *et al*. 1996*b*; Bruce *et al*. 1997; Hill *et al*. 1997*a*; Asante *et al*. 2002). There has been widespread dietary exposure of the UK population to BSE prions; the total cattle epidemic has been estimated at approximately 2 million infected animals (Donnelly *et al*. 2002). Cattle BSE has also been reported in most member states of the European Union, Israel, Switzerland, Canada, the USA and Japan. To date, approximately 170 vCJD patients have been recognized in the UK and approximately 30 cases from other countries including France, Italy, The Netherlands, Ireland, Spain, Canada, Japan and the USA. Predictions of the eventual size of the vCJD epidemic, based on mathematical modelling and the clinical cases of vCJD to date, though varying widely, have suggested that the total epidemic may be relatively small (Ghani *et al*. 2003). However, there are many uncertainties, notably with respect to major genetic effects on incubation period (Lloyd *et al*. 2001). Moreover, these models have not estimated the number of *infected* individuals, which may prove to be the most important aspect of the vCJD epidemic. We do not know the human infectious or lethal dose of BSE prions by the oral or any other route, the cumulative effect of multiple dosing or whether, in addition to preclinically affected individuals, there are subclinical carrier states of prion infection in humans, as recognized in several animal models (Hill *et al*. 2000; Asante *et al*. 2002; Thackray *et al*. 2002; Hill & Collinge 2003). If so, this will prove to be a significant problem for secondary transmission of vCJD via medical and surgical procedures performed on asymptomatic individuals incubating the disease. Importantly, the pathogenesis of vCJD differs substantially from that of other forms of CJD in that, unlike classical CJD, lymphoreticular tissues, including lymph nodes, are extensively (and uniformly) involved with PrP^Sc^ levels in tonsil up to 10% of those seen in the brain (Wadsworth *et al*. 2001). Four patients have now been identified with vCJD prion infection from a small cohort of individuals (currently approx. 23) known to have received blood transfusion from a donor who subsequently developed vCJD, suggesting that the transmission of vCJD prion infection by this route may be relatively efficient (Llewelyn *et al*. 2004; Peden *et al*. 2004; Wroe *et al*. 2006; Wroe *et al*., unpublished data). A retrospective study of archived surgical lymphoreticular specimens (largely appendix) reported 3 out of 12 674 positive for PrP immunoreactivity and estimated prevalence of infection at 237 per million (95% confidence interval 49–692 per million) (Hilton *et al*. 2004). Appendix appears to be a less sensitive reporter of vCJD prion infection than tonsil (Joiner *et al*. 2002), and this retrospective study may therefore have underestimated prevalence. A prospective national-scale anonymous screen of discarded tonsillectomy tissue for PrP immunoreactivity was announced by the Chief Medical Officer and is now underway to try to better estimate prevalence of asymptomatic infection with vCJD prions in the community. These concerns and uncertainties, particularly with respect to the possibility of extremely prolonged incubation periods of BSE in humans, have renewed interest in kuru, which remains our only experience of a major epidemic of human prion disease, and from which there is much to be learned of direct relevance to vCJD.

Kuru reached epidemic proportions in the people of the Fore linguistic group and the neighbours with whom they intermarried, living in the Eastern Highlands of Papua New Guinea (Zigas & Gajdusek 1959). Local oral history, recorded from when the disease was first studied by western medicine in 1957, dated the onset of the first cases to at least as far back as the 1920s. Kuru predominantly affected women and children (of both sexes), with only 2% of cases in adult males (Alpers 1987). Kuru was always fatal and, in affected villages, was the commonest cause of death among women. It was the practice in these communities to engage in the consumption of dead relatives as a mark of respect and mourning (transumption). Males over the age of 6–8 years participated little in mortuary feasting, which explains the differential age and sex incidence. It is hypothesized that the epidemic originated from the consumption of a sporadic CJD case occurring in the region some decades earlier (Alpers & Rail 1971). Epidemiological studies provided no evidence for vertical transmission, since most of the children born after 1956 (when endocannibalism had effectively ceased) and all of those born after 1959 of mothers affected with or incubating kuru were unaffected (Alpers 1987; Collinge *et al*. 2006). From the age of the youngest affected patient, the shortest incubation period is estimated as 5 years, although it may have been shorter, since the time of infection was usually unknown. The age of onset of disease in kuru has ranged from 5 to over 60 years. The mean clinical duration of illness is 12 months with a range of 3 months to 2 years, though in a few atypical cases it may be much longer; the course tends to be shorter in children (Alpers 1964*c*).

The central clinical feature is progressive cerebellar ataxia. In sharp contrast to classical CJD, dementia is reported to be much less prominent and may be absent, although in the later stages many patients have their faculties obtunded (Alpers 1987). The occasional case in which gross dementia occurs was thought to be in marked contrast to the clinical norm. Kuru does not appear to have changed in its features at different stages of the epidemic reported to date and has a well-characterized clinical progression with a distinct prodrome and three clinical stages. Kuru typically begins with prodromal symptoms consisting of headache, aching of limbs and joint pains, which can last for several months. This is followed by the onset of the first, or ambulatory, stage. Kuru was frequently self-diagnosed by patients at the earliest onset of unsteadiness in standing or walking, or of dysarthria or diplopia. At this stage, there may be no objective signs of disease. Gait ataxia, however, worsens and patients develop a broad-based gait, truncal instability and titubation. A coarse postural tremor is usually present and accentuated by movement; patients characteristically hold their hands together in the midline to suppress this. Standing with feet together reveals clawing of toes to maintain posture. This marked clawing response is regarded as pathognomonic of kuru. Patients often become withdrawn at this stage and occasionally develop a severe reactive depression. The prodromal symptoms tend to disappear. Astasia and gait ataxia worsen and the patient requires a stick for walking. Intention tremor, dysmetria, hypotonia and dysdiadochokinesis develop. Although eye movements are ataxic and jerky, nystagmus is rarely seen. Strabismus, usually convergent, may occur, particularly in children. This strabismus does not appear to be concomitant or paralytic and may fluctuate in both extent and type, sometimes disappearing later in the clinical course. Photophobia is common and there may be an abnormal cold sensitivity with shivering and piloerection even in a warm environment. Tendon reflexes are reduced or normal and plantar responses are flexor. Dysarthria usually occurs. The mean clinical duration of the first stage is approximately eight months and correlates closely with total duration (Alpers 1964*c*). Ataxia progresses and the loss of ability to walk without support marks the transition from the first (ambulatory) stage to the second (sedentary) stage. Attempted walking with support leads to a high-steppage, wide-based gait with reeling instability and flinging arm movements in an attempt to maintain posture. Hyperreflexia is seen, although plantar responses characteristically remain flexor with intact abdominal reflexes. Clonus is often marked though invariably short lived. Athetoid and choreiform movements and parkinsonian tremors may occur. There is no paralysis, although muscle power is reduced. Obesity is common at this stage but may be present in early disease associated with bulimia. Characteristically, there is emotional lability and there may be euphoria with inappropriate laughter, which has led to the disease being referred to as the ‘laughing death’. There is no sensory impairment. In sharp contrast to CJD, myoclonic jerking is rarely seen. This stage typically lasts approximately two to three months. When truncal ataxia reaches the point where the patient is unable to sit unsupported, the third or recumbent stage is reached. Hypotonia and hyporeflexia develop and the terminal state is marked by a flaccid muscle weakness. Plantar responses remain flexor and abdominal reflexes intact. Progressive dysphagia occurs and patients become incontinent of urine and faeces. Inanition and emaciation develop. Transient conjugate eye signs and dementia may occur. Primitive reflexes develop in occasional cases. Brainstem involvement and both bulbar and pseudobulbar signs occur. Respiratory failure and bronchopneumonia or septicaemia eventually leads to death. The tertiary stage typically lasts one to two months, though prolongation of the terminal moribund state is not uncommon.

Transmission of CJD in western societies has occurred by accidental inoculation with human prions as a result of medical and surgical procedures. Such iatrogenic routes, which may be considered a western counterpart of cannibalism, include the use of inadequately sterilized neurosurgical instruments, dura mater and corneal grafting, and intramuscular injection with human cadaveric pituitary-derived growth hormone or gonadotrophin. Most recently, cases of iatrogenic infection with vCJD prions arising from blood transfusion have emerged (Llewelyn *et al*. 2004; Peden *et al*. 2004; Wroe *et al*. 2006). Interestingly, cases arising from intracerebral or optic inoculation manifest clinically as classical CJD, with a rapidly progressive dementia, while those resulting from peripheral inoculation, most notably following cadaveric pituitary-derived growth hormone exposure, typically present with a progressive cerebellar syndrome, and are in that respect somewhat reminiscent of kuru. Therefore, one possibility is that the route of infection is an important determinant of kuru phenotype, although prion strain type and host genetic background are also likely to be important factors.

Kuru was extensively studied at its peak in the late 1950s and the early 1960s and monitoring was continued through the Institute of Human Biology (later, Papua New Guinea Institute of Medical Research (PNGIMR)) from the late 1960s and by the field team established by M.P.A. in the early 1970s, later incorporated into the field team of the PNGIMR, which has maintained surveillance continuously since then. We strengthened active surveillance in 1996 and aimed to study all kuru patients until the end of the epidemic. In particular, we wished to determine the maximum incubation periods possible in human prion infection, now estimated to be more than 50 years (Collinge *et al*. 2006). The effective prohibition of endocannibalism by the Australian authorities in the mid-1950s led to the abrupt cessation of transmission of kuru, which allows a unique opportunity to investigate the incubation period, a key parameter in human prion disease. We investigated whether any remaining sources of infection persisted, including any evidence of maternal or other routes of kuru transmission, and sought to compare the clinical and other diagnostic features of kuru with the other acquired human prion diseases, iatrogenic CJD and vCJD, which show a number of similarities to kuru.

Kuru also provides a unique opportunity to characterize genetic susceptibility and resistance factors and to gain insights into the peripheral pathogenesis of orally acquired prion disease in humans. Genetic susceptibility is significant in both the sporadic and acquired forms of human prion disease. There is a common polymorphism in human PrP, with either methionine or valine present at residue 129. In Europeans, approximately 38% are homozygous for the more frequent methionine allele, 51% are heterozygous and 11% homozygous for valine. The large majority of sporadic CJD occurs in individuals homozygous for this polymorphism (Palmer *et al*. 1991). This susceptibility factor is also relevant in the acquired forms of CJD, most strikingly in vCJD, where all clinical cases studied to date have been *PRNP* codon 129 methionine homozygous (Collinge *et al*. 1991, 1996*a*). The *PRNP* codon 129 genotype has a significant effect on kuru incubation periods and susceptibility, and the most elderly survivors of the kuru epidemic are heterozygous (Cervenakova *et al*. 1999; Mead *et al*. 2003). The marked survival advantage for those heterozygous at codon 129 provides a powerful basis for selection pressure in the population affected by kuru. Remarkably, an analysis of the worldwide haplotype diversity and allele frequency of *PRNP* coding and non-coding polymorphisms suggests that balancing selection at this locus is not restricted to the Fore but is much older and more geographically widespread. Evidence for balancing selection (where there is more variation than expected in a gene due to heterozygote advantage) has been demonstrated in only a few human genes. Given recent biochemical and physical evidence of cannibalism on five continents, one explanation is that cannibalism resulted in a series of prion disease epidemics in human prehistory, thus imposing balancing selection on *PRNP* (Mead *et al*. 2003).

We also sought to provide further data for the accurate epidemiological modelling of the kuru epidemic and to document mortuary feast practices and traditional beliefs of the aetiology of kuru by interview of surviving participants and other members of the Fore community; here we report our clinical studies and investigation of *PRNP* in recent kuru patients.

## 2. Material and methods

### (a) Research ethics

The clinical and laboratory studies were approved by the Medical Research Advisory Committee of the Government of Papua New Guinea and by the local research ethics committees of St Mary's Hospital and the Institute of Neurology and National Hospital for Neurology and Neurosurgery in London. Critically important from both the ethical and operational aspects was the full participation in the project of the communities involved. This was established and maintained through discussions with village leaders, communities, families and individuals. The field studies followed the principles and practice of the PNGIMR, in collaboration with which this study was performed.

### (b) Kuru surveillance and clinical studies

A field base and basic laboratory for sample processing and storage was established in the village of Waisa in the South Fore. A team of local kuru reporters communicated to the field base the details of any person suspected of suffering from kuru. The large majority of these suspect cases would not be confirmed as kuru on subsequent investigation by the field team or the visiting Medical Research Council (MRC) Unit medical staff; 50 suspect cases investigated during this period proved not to have kuru. The field team comprised MRC, PNGIMR and local staff, and undertook regular field patrols throughout the kuru-affected area, which includes the North and South Fore, Keiagana, Kanite and Gimi linguistic groups. The fieldwork involved walking in arduous terrain in poor weather conditions at high altitude in the Eastern Highlands Province. Roads were frequently impassable and getting supplies to the field, and samples from it, often needed heavy-vehicle rescue equipment and a large team. Security issues intermittently affected our ability to travel to and from the field site. Regular field neurological examinations were performed when possible (by J.C., M.P.A. or D.T.) and patients' clinical features were documented by filming on video and by still photography. Case histories and exposure to mortuary feasts were documented. Venous blood samples were taken for molecular genetic studies from patients and, where possible, from family and community members.

### (c) Tonsil biopsy

With informed consent from the patient and his family, tonsil biopsy was performed at Goroka General Hospital by one of us (A.F.), with assistance from local medical staff using disposable instruments, as previously described (Hill *et al*. 1999). A portion of the tonsil tissue was fixed in 10% formol saline and the rest shipped frozen on dry ice to London for analysis. PrP immunohistochemistry was performed using anti-PrP monoclonal antibody ICSM 35 (D-Gen Ltd, London) and Western blot analysis for PrP^Sc^ performed as previously described (Wadsworth *et al*. 2001).

### (d) Molecular genetic studies

The methodology used was as previously described (Mead *et al*. 2001). In brief, genomic DNA was extracted from venous blood and the complete coding sequence of the prion protein gene (*PRNP*) determined by the direct sequencing of the polymerase chain reaction (PCR)-amplified open reading frame. Restriction endonuclease digestion of PCR amplicons was used to analyse the apolipoprotein E gene (*APOE*) and the prion-protein-like protein gene (*PRND*). The ABI SDS 7000 sequence detection system was used for the allelic discrimination of *PRNP* codon 129 and *PRNP* haplotypes. HLA-DQB1 alleles were determined by the automated fluorescent sequencing of PCR amplicons using the Amersham MegaBACE DNA analysis system.

## 3. Results

**Figure 1:**
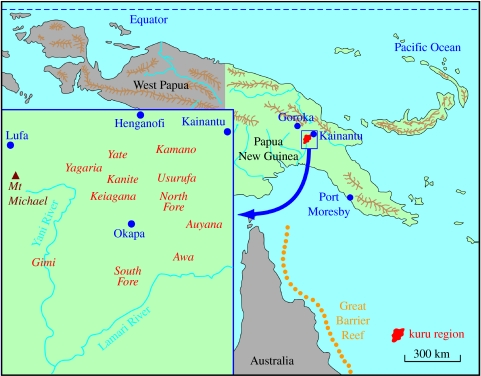
The area and linguistic groups historically affected by kuru in the Eastern Highlands Province of Papua New Guinea.

**Figure 2:**
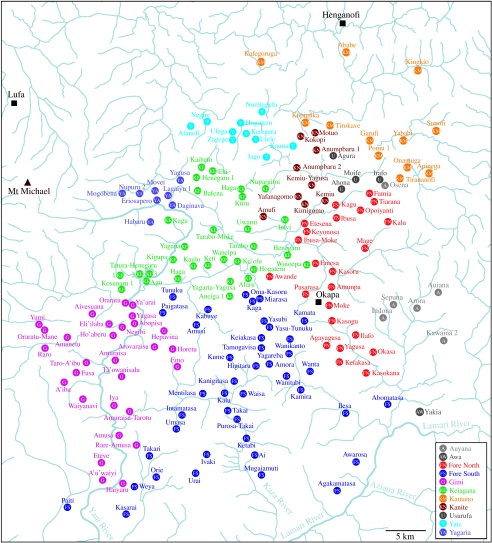
Map showing the location of all villages that have had a history of kuru. Of the 172 village names on the map, 155 appear in the database of patients recorded since kuru surveillance began in 1957; however, only 145 of these villages have had confirmed cases during this time. Over 80% of all the recorded cases have occurred among the Fore linguistic group (South and North Fore combined). Map derived from Gajdusek *et al*. (1961), Alpers (1964*a*), Alpers and the Kuru Surveillance Team (Alpers 2005) and the kuru database (created by M.P.A., Judith Farquhar, Steven Ono and D. Carleton Gajdusek, and maintained by M.P.A.).

Since July 1996 to the end of June 2004, we identified 11 kuru patients. The sex, age of onset and duration of disease of all patients are given in table 1. Age is not usually accurately known in these communities but can be reliably estimated by reference to family relationships and historical events (Alpers 1964*a*). The area and linguistic groups historically affected by kuru are illustrated in figure 1. Figure 2 locates all the villages that have had a history of kuru and shows the shape and extent of the kuru region. All patients identified in the current study were from the South Fore, kuru now having disappeared from the North Fore and other linguistic groups to the north. The 11 patients were from 10 villages, as shown in Collinge *et al*. (2006); there were two patients from Kamira, which since 1957 has had more patients than any other village.

Of the 11 patients, four were female and seven male. The age of onset ranged from 46 to 63 years. The ages at death included two in their 40s, five in their 50s and four in their 60s. These distributions are strikingly different from those found when kuru was first analysed (Zigas & Gajdusek 1959; Gajdusek *et al*. 1961; Alpers 1964*a*; Alpers & Gajdusek 1965): 60% of the cases in adult women, with the remainder mostly in children and adolescents of both sexes. In 1957–1958, the proportion of cases in adult males was 2%; this percentage gradually rose as the disease died out in children and adolescents, but the numbers of adult male deaths from kuru were never high (Alpers 1987). The demography of the South Fore in 1962 (Alpers & Gajdusek 1965) shows that in a population of 7224, 6% of males and 4% of females were aged 50 years or more. These percentages are low but consistent with the life expectancy of a traditional society; the difference is one example of the effect that kuru had on the Fore demography (by comparison, the corresponding figures for the 2001 population of the UK are 31 and 35%). The major perturbation of the demography by kuru was in the male:female ratio of the population: 1.64 in the South Fore in 1962 (in the UK it is 0.95). Despite the relatively small proportion of older people in the population, in the 7-year period of 1957–1963, out of 1254 deaths from kuru, 203 were in females aged 40 years or more (Alpers 1979), including 54 in their 50s and 19 in their 60s. In striking contrast, there were only two male deaths from kuru in this age group (Alpers 1979). Since both of these deaths occurred in 1962, all males who died of kuru before 1962 were aged less than 40 years (unpublished data from the kuru epidemiological database).

The duration of the disease from the onset of kuru to death varied in the 11 patients from 10 to 25 months. The mean was 17 months and standard deviation (s.d.) 5 months. This is consistent with earlier findings (Alpers 1964*c*) and the established range of 3–23 months found in 1962–1963, except that there were no cases of short duration. The mean is consequently higher than the 12.5 months (s.d. 5 months) previously found; however, since that study included all ages and showed that duration tended to lengthen with age, the present finding in patients with a mean age of onset of 55 years is not exceptional.

The clinical features of contemporary kuru patients are exemplified by the following case histories.

### (a) Patient AKA

Male patient of age estimated at 48 years was first examined in June 1997. At that time, he had a seven-month history of feeling inappropriately cold, first in his lower limbs and then becoming generalized. He had noted progressive unsteadiness in walking during this period. There was no limb or joint pain, stiffness or weakness. He described frequent headaches with their onset in the afternoon. At this stage, the patient attributed his illness to chronic malaria, although relatives and community members suspected kuru. He was otherwise well and taking no medication. He was born in the South Fore and spent his childhood there in two villages severely affected by kuru. Local oral history from older members of the community confirmed his participation in multiple mortuary feasts as a child with his mother. His father had died of an arrow injury and his mother died of kuru in 1953 at the age of 23. He was then adopted and lived with his foster parents until 1962 when he lived in the male house until its closure in 1972. He worked on a copra plantation in East New Britain Province before returning to the highlands in 1976 and worked as a storekeeper in Goroka until 1980. He then worked for the local coffee company until the onset of his illness. He had a sister who was well with six children. He was married with one adopted child.

On examination in his village, he was alert, orientated and fully cooperative with appropriate affect. No pathological laughter was noted. He sat with hands held tightly together, suppressing involuntary movements. There was titubation. He was clinically euthyroid. Eye movements were full without diplopia and visual pursuit was smooth without nystagmus. There was no facial weakness and the pupils were symmetrically responsive to light and accommodation. He was restless with some choreiform movements of upper limbs. There was no rest tremor or fasciculation. Tone and power were normal in the limbs and Romberg's sign was negative. Tendon reflexes were symmetrical and not brisk and plantar responses flexor. He had moderate impairment of finger–nose coordination in the upper limbs and dysdiadochokinesis. Sensation was intact to touch and proprioception was intact. His gait was somewhat wide based and tandem gait markedly impaired. He was unsteady on one foot with marked toe clawing. A clinical diagnosis was made of ambulatory (stage 1) kuru. *PRNP* analysis demonstrated normal coding sequence and the codon 129 genotype was methionine/valine heterozygous.

Following informed consent, the patient was transferred to Goroka General Hospital for further review and tonsil biopsy. When examined in November 1997, he remained in stage 1 (ambulatory) kuru. Despite full insight into his condition and prognosis, his mood was elevated with some evidence of euphoria. His astasia, gait ataxia and upper limb incoordination had worsened, but otherwise his clinical signs had not significantly changed over the intervening five months. He was still able to walk without support and noted himself the unusually slow progression. Tonsil biopsy was performed.

By February 1998, he had lost the ability to walk without support and, 15 months after onset, therefore moved into the second (sedentary) stage of the disease. This progressed over the next three months, and, by May 1998, he was unable to sit up without support and was considered to have entered the third (recumbent) stage of kuru; two months later, he died following a total illness duration of 20 months. No autopsy was performed.

### (b) Patient AYY

Male patient of age estimated at 60 years when examined in his village in February 2000. His symptoms began in June 1998 when he noted interphalangeal foot pain on walking, initially on the right and then bilaterally. He noted unsteadiness in walking and complained of cold feet and shivering despite warm conditions. There was no headache. By December 1999, he was unable to walk without active support and had developed poor memory and was unable to recognize his house or names of close relatives even when present. There was a past history of malaria. He was taking no medication. He was born in 1940 and lived in the South Fore continuously throughout the period endocannibalism was practised. Local oral history from older members of the community confirmed his participation in multiple mortuary feasts as a child. His brother died of kuru in 1952 at the age of 17.

On examination, he was thin. Cognitive examination was limited by linguistic and cultural factors. However, he was orientated to person, but not to time or place. He was unable to recall any of three objects. He did not have significant problems with praxis and was able to copy hand movements. There was cerebellar dysarthria. Eye movements were full without diplopia, but pursuit was broken. There was no nystagmus and pupils responded to light and accommodation. Cranial nerves were otherwise intact. There was no wasting of the limbs and no rest or postural tremor, myoclonus or other involuntary movements. Tone was normal and power full in the limbs. There was marked cerebellar ataxia of all four limbs with gross intention tremor and marked dysdiadochokinesis. Tendon reflexes were present and symmetrical but present at the ankles only on reinforcement. Plantar responses were flexor. Vibration sense was absent in both the lower limbs to the hips bilaterally but pinprick was intact with hyperaesthetic arms. Joint position sense was intact. He was able to sit up without assistance but walking required the support of two assistants. There was marked truncal ataxia and a broad-based gait. Toe clawing was absent. Cardiovascular examination was normal with a blood pressure of 100/70 without postural drop. There was no organomegaly. A clinical diagnosis of second stage (sedentary) kuru was made. *PRNP* analysis was performed and demonstrated normal coding sequence and the codon 129 genotype was methionine/valine heterozygous. He died at the age of 60 following a total disease duration of 22 months.

### (c) Patient WKW

Male patient of age 57 years when examined in the village in February 2000. He developed headache and abdominal pain in January 1999. There were two episodes of occipital headache with mandibular and right ear pain. He also developed joint pains, starting in the knees and associated with stiffness and then also involving the upper limbs and the hands during the second episode of headache. He developed unsteadiness of gait and began to lose weight. In October 1999, he developed diplopia, dysphasia and experienced memory difficulties. By January 2000, he was unable to walk unsupported. At the time of examination in February 2000, family members reported that he was forgetting the identity of close family members, but was able to recognize them when present. He was taking no medication. There was no past medical history of note. He was resident in the South Fore throughout his childhood. There was no immediate family history of kuru. Local oral history from older members of the community confirmed his participation in multiple mortuary feasts as a child.

On examination, he was able to understand requests in Tok Pisin (the lingua franca of Papua New Guinea) but reverted to his native Fore language in replies. Cognitive testing was limited by linguistic and cultural factors. However, he was orientated to name, but not to time or place. He was unable to recognize his own house in the village. He was able to repeat and recall two of three objects. He was unable to count or copy a drawing of a flower or house, although such cognitive testing may be of limited validity in a Fore individual of this age. He was able to complete a three-stage command and name the local representative and missionary at a nearby larger village. He was able to copy simple hand movements and a salute. There was marked perseveration. Affect appeared normal with appropriate laughter. There was no astereognosis; he was able to correctly name coins, a pen and various vegetables by hand with eyes closed. There was bilateral pout reflex, but palmomental and grasp reflexes were absent. He had a brisk jaw jerk. He was examined in bright light in the village and pupils were small but symmetrical. There was an equivocal light response but definite response to accommodation. Eye movements were full, but pursuit jerky. There was no nystagmus or diplopia and visual fields were intact to confrontation. There was reduced left trigeminal sensation to touch and pinprick and reduced left corneal response. There was no facial weakness, and hearing, palatal movement and tongue movements were normal. Limbs were generally thin with fasciculation of medial thighs bilaterally. There was no wasting of the small muscles of the hand. There was no myoclonus, rest or postural tremor. There was no dystonic posturing but there was marked clawing of the toes while standing. There was extrapyramidal increase in tone in both arms, left greater than right. Limb power was normal with the exception of mild weakness (4/5) in finger abduction. There was bilateral cerebellar ataxia of the upper and lower limbs and more marked truncal ataxia. Tendon reflexes were generally depressed but obtained on reinforcement except at ankle. Plantar responses were flexor. He was able to sit up without assistance but required two assistants to walk. Gait was then broad based and symmetrical. Romberg's test was negative standing and sitting. There was loss of vibration sense in both the lower limbs to the hips but joint position sense and pinprick were preserved. Cardiovascular examination was normal with a blood pressure of 90/70 without postural drop. There was no organomegaly. A clinical diagnosis of second stage (sedentary) kuru was made. *PRNP* analysis was performed and demonstrated normal coding sequence and the codon 129 genotype was methionine/valine heterozygous. He died at the age of 57 following a total disease duration of 17 months.

### (d) Patient MAA

Female patient aged 56 years when examined in the village in August 2000. Her symptoms began in April 1999 with leg pain, joint pain in her knees and difficulty walking; she had been seen briefly the following month as possibly suffering from kuru. Her speech and vision had been normal. There was no past medical history of note and she was taking no medications. Her father had been killed by ‘friendly fire’ during clan fighting, which led to her mother, then pregnant with her, fleeing to a neighbouring linguistic group, where she was born and grew up. She visited her paternal village in the South Fore with her mother during childhood and moved back there at her marriage. Her mother died in the adoptive community but the cause was not known to her or her relatives. She had two husbands, both of whom died, one of an unknown illness and the other probably of liver cancer. She had seven children, all of whom are alive and well, and four grandchildren.

On examination, she was cheerful and responded well to questioning. She was mildly euphoric. There was no emotional lability or gross cognitive impairment. There was no dysphasia. Cranial nerves were intact. Eye movements were not jerky and there was no diplopia or nystagmus. Titubation was present. The limbs were hypertonic with jerky rigidity, pipe-like in quality, noted equally in both arms. There was no dysmetria in the upper limbs, intention tremor or dysdiadochokinesis. Heel–shin testing was performed well until the feet came together in the midline. Tendon reflexes were uniformly diminished and plantar responses flexor. She showed truncal instability but no Rombergism. No sensory abnormality was detected and proprioception was normal.

Her gait was wide based and severely ataxic but she was able to walk independently if supported by a stick. There was marked astasia with clawing of her toes. A clinical diagnosis of stage 1B kuru was made (stage 1 can be subdivided into 1A where the patient can walk entirely unaided, and 1B if a stick is necessary to walk). At the very end of the first stage of kuru, she showed clear evidence of fully established midline cerebellar disease and some extrapyramidal features. Unusually, there were no signs at that time of neocerebellar involvement. The first stage of kuru lasted 17 months. *PRNP* analysis demonstrated normal coding sequence and the codon 129 genotype was methionine/valine heterozygous. Her disease progressed quite rapidly over the next five months until she died in January 2001. Autopsy was not performed.

### (e) Patient KAW

Male patient aged 58 years when seen in September 2001. He had experienced episodes of pain and weakness in the legs for several years, which had made walking difficult; these had responded to local treatment, which consisted of counterirritants, bloodletting from the calf and herbal medicines, but then recurred. He also had headaches and pain in the neck, arms and thoracic and abdominal muscles. He was convinced that these were ‘attacks of kuru’ and that he was having such an attack when seen. When examined, he was complaining of pain but was voluble and relaxed, with no objective evidence of pain. He had frequent fasciculations in his calf muscles. His posture was steady, his stance firm and his gait normal. He declined formal examination. Plasma electrolyte levels, measured in the regional hospital in Goroka, were normal. He was born in 1943 and lived in the South Fore continuously during the period when traditional mortuary feasts were held. His mother died of kuru in 1965, and local oral history from older members of the community confirmed his participation in multiple mortuary feasts as a child.

During the next month, he went travelling within the highlands. On his return, he complained of unsteadiness of gait, which slowly worsened. During the next 10 months, he followed a progressive course with worsening cerebellar ataxia typical of kuru, and by the end of August 2002 this had entered the second (sedentary) stage. After three months, he was unable to sit without support and became recumbent (stage 3 of kuru). He alternated between periods of confusion and lucidity. He still showed occasional fasciculations. He was examined about a month after he became recumbent. He was well nourished and in no pain. He was lucid and able to converse sensibly. He was calm and rational, with some flattening of affect. He had insight into his disease and said that his only worry was what would happen to his children after he died. He was continent of urine and faeces and had no pressure sores. He had severe photophobia. He had pronounced cerebellar dysarthria. Eye movements were full but jerky; there was no nystagmus. Cranial nerves were otherwise intact. There were no abnormal facial movements or dysconjugate eye movements. He was able to sit up only with external support and had marked truncal instability. His hands were held clasped in front to suppress involuntary postural tremors. No fasciculations were seen at this time. He had plastic, jerky rigidity of all four limbs. His legs were weak. Power in the arms and hands was also reduced to grade 3/5. Tendon reflexes were diminished or absent; plantar responses were flexor. There was dysmetria in finger and hand movements, intention tremor in finger–nose alternation and dysdiadochokinesis. He performed all these tests without hesitation or confusion.

Thereafter, his physical condition progressively declined, with increasing weakness and disintegration of movement. He became incontinent, developed pressure sores in his sacral area, in both buttocks and on both heels, and dislocated his left hip. He ate very little and subsisted largely on sips of water. He was unable to speak but continued to make eye contact and followed people with his eyes. He persisted in this state for another four months. In April 2003, he lost consciousness for a day but by the next day had regained the visual awareness of his surroundings and again made eye contact, and the only movement was of his eyes. He remained in this state for 3 days. He was totally moribund the next day and died on the following day. *PRNP* analysis demonstrated normal coding sequence and the codon 129 genotype was methionine/valine heterozygous. Autopsy was performed and the diagnosis was neuropathologically confirmed. A detailed description of the autopsy findings is given in Brandner *et al*. (2008).

### (f) Analysis of tonsil biopsy

**Figure 3:**
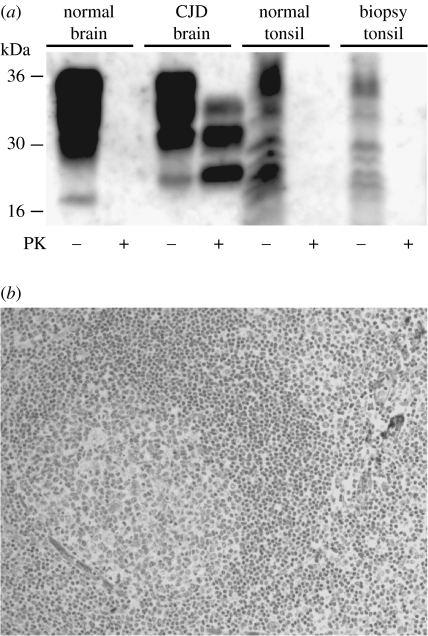
Tonsil biopsy from patient AKA. (*a*) Immunoblot analysed with anti-PrP monoclonal antibody 3F4 and enhanced chemifluorescence. Ten-microlitre aliquots of 10% normal human brain homogenate or 10% CJD brain homogenate and 20 μl aliquots of 10% normal human tonsil homogenate or 10% tonsil biopsy homogenate from a patient with kuru were analysed before (PK−) or after (PK+) proteinase K digestion. (*b*) Immunohistochemical analysis with anti-PrP monoclonal antibody ICSM 35. The photomicrograph shows a lymphoid follicle with abnormal PrP immunoreactivity being absent.

It is of considerable interest as to whether kuru, transmitted by dietary prion exposure, is associated with tonsillar prion infection, as seen in vCJD, which is also thought to be transmitted by dietary exposure, but not in other forms of human prion disease. Tonsil biopsy tissue was examined by PrP immunohistochemistry using anti-PrP monoclonal antibody ICSM 35 and no abnormal staining of follicular centres or other areas was seen, in sharp contrast to vCJD tonsil (Wadsworth *et al*. 2001; figure 3*b*). Western blot analysis did not demonstrate PrP^Sc^ and remained negative following high-sensitivity analysis involving NaPTA precipitation from tissue homogenate to concentrate any PrP^Sc^ present (Wadsworth *et al*. 2001; figure 3*a*).

### (g) Incubation period estimation

These results have been reported by Collinge *et al*. (2006). They are, however, presented and discussed here in the context of the local mortuary practices and epidemiological and clinical findings. The transmission of the infectious prion of kuru occurred through the endocannibalism (transumption) of dead relatives at mortuary feasts held in their honour; our understanding of this is based on a conjunction of experimental, epidemiological and human behavioural evidence (Alpers 1968). The practice was important to the people as a way of respecting their dead, but it was rigorously proscribed by the Australian government officers in one of their first acts of administrative control after making contact with the people. The Okapa Patrol Post was established in 1954. Public consumption of the dead ceased almost immediately and compliance was ensured by the police force responsible for the subdistrict. By 1956, endocannibalism was suppressed. There were reports of surreptitious eating of the dead in remote hamlets for some years afterwards, but by 1960 the practice had effectively ended; though after this time a few dead bodies may have been exhumed and partially eaten by older women determined to maintain their proper respect for the dead, this would not have had any epidemiological significance since such women would have had multiple previous exposures. Epidemiological surveillance for kuru began in 1957 and has been continued ever since (Alpers 2005). Owing to the wide geographical extent of the families involved in a feast, secret feasting with whole families taking part would not have gone undetected. The communities of the North Fore, who had been the first of the Fore people to lose their traditional practices in the wake of Australian administrative control, ended their mortuary feasting at the beginning of the decade or earlier; kuru is no longer present in this area. The latest year of birth recorded for any kuru patient is 1959. From this combination of evidence, we can conclude that transmission through the traditional mortuary practices had ceased by 1960. This enables us to obtain a measure of *minimum* incubation period as the time between 1960 and the date of onset of kuru. The range of minimum incubation period in the 11 patients was 34–41 years (Collinge *et al*. 2006).

In males, after the age of 6–8 years, boys were taken from their mother and brought up in the men's house. From then on, they were exposed only to the same risk as adult males, who participated little in feasts and did not eat the brain, by far the most infectious organ in kuru. This can explain why in 1957–1958 adult males contributed only 2 per cent to the total number of kuru cases and, from what we now know about the incubation period, most of these adult male cases would have been from transmissions in their childhood. This also explains why all males dying of kuru before 1962 were aged less than 40 years: those older would have been aged 6–8 years before 1930, when the epidemic was still slowly building up and their childhood risk of exposure to infection consequently small (females, by contrast, were exposed at all ages throughout the epidemic). From the male ages of exposure, we can estimate the *likely* minimum incubation period of the males in the recent group of patients described here as extending from the year in which they turned 7 to the year of onset (and it could possibly be up to 7 years longer). The range of likely incubation period in the seven males in table 1 is 39–56 years. Compared with the UK population, the people of the kuru region, even now, have a relatively low life expectancy; hence, many long incubation cases of kuru would have died of some other cause and would not have been detected. The proportion of such cases relative to those of shorter incubation period will therefore be underestimated in the study described here. Nevertheless, what we have been able to demonstrate is the fact that incubation periods of this length do occur in human prion diseases.

### (h) Genetic analysis

Results of genetic analysis in these patients have been reported elsewhere (Collinge *et al*. 2006) and are summarized here and compared with those in the healthy South Fore population. DNA was available for analysis in 10 out of 11 kuru patients and 8 out of 10 were heterozygous at polymorphic residue 129 of *PRNP* (table 1). Allele frequencies at *PRNP* 129 in 140 unrelated healthy South Fore were 129M (methionine; 48%) and 129V (valine; 52%). Although the majority of the long incubation kuru cases were heterozygous, as expected from the known protective effect of this polymorphism, this distribution was not statistically different from the current normal population frequencies (*Χ*^2^-test, *p*=0.22). However, a comparison of clinical duration of illness against codon 129 genotype is interesting in that the two homozygous patients have the shortest durations (10 and 12 months) when compared with the eight heterozygotes. The clinical durations for the two homozygous patients (10 and 12 months) fall 4.2 and 5.2 s.d. below the mean clinical duration for heterozygous patients (mean 20.4 months and s.d. 2.0 months). Although formal statistical tests are not appropriate with only two observations, these data support a modifying effect of codon 129 genotype.

The *PRNP* B haplotype has been associated with susceptibility to sporadic CJD in the UK (Mead *et al*. 2001) and *PRNP* haplotyping was carried out in these patients and in the normal Fore population. Nine single nucleotide polymorphisms within 25 kb of *PRNP* codon 129, identified in the European population, may be used to predict *PRNP* haplotypes. Two common haplotypes are found in the South Fore: 129M is associated with haplotype F (haplotype frequency 40%) and 129V with haplotype A (haplotype frequency 52%). Haplotype B, known to be a common variant in Europe and overrepresented in sporadic CJD patients, was found at a 3 per cent frequency in the South Fore. Albeit from the analysis of a small sample size, the distribution of *PRNP* haplotypes in the long incubation kuru patients was not significantly different from that in the healthy South Fore population (*Χ*^2^-test, *p*=0.85).

As we have previously reported, allele frequencies at the *PRND* codon 174 polymorphism, *APOE* and HLA-DQB1 in these long incubation kuru patients were not significantly different from those in the current healthy South Fore population, although, clearly, the number of patients analysed was small (Collinge *et al*. 2006).

## 4. Discussion

Epidemiologically, kuru is defined by its essential clinical features of cerebellar ataxia, its subacute, progressive course and its fatal outcome (Alpers 2005). Neuropathological confirmation was obtained in a small proportion of cases in the past, but this was of major significance in characterizing kuru as a transmissible spongiform encephalopathy (in fact, it was the key to developing the concept of such a disease entity, once transmission to the chimpanzee had been achieved; Fowler & Robertson 1959; Hadlow 1959; Klatzo *et al*. 1959; Beck *et al*. 1966; Gajdusek *et al*. 1966). All the 11 recent patients in the present series were determined through the same process of epidemiological surveillance as had been conducted in the past and the results are therefore entirely consistent with case ascertainment throughout the kuru epidemic. In one case, an autopsy was obtained (which is a higher proportion than was possible to achieve in the past). This gave neuropathological confirmation of kuru, which is important in validating the epidemiological and clinical findings reported here. The autopsy enabled many other studies to be undertaken, which will be reported separately.

While the clinical phenotype of these recent kuru cases lies within the spectrum described for earlier cases, there are some unusual features worthy of comment. The average duration of illness (17 months) is longer than that reported overall for earlier cases (12.5 months) but is consistent with previous findings of longer duration in older patients; in the previous study that had specifically addressed this question, in patients aged 40 years or more the mean duration was 18 months (Alpers 1964*c*). Interestingly, however, the two patients homozygous at *PRNP* 129 had significantly shorter clinical durations than the heterozygous patients. Although this should be interpreted with caution in view of the small numbers, it is possible that the high mean duration overall in these cases of long incubation period reflects an effect of *PRNP* heterozygosity on disease duration. In sharp contrast to sporadic CJD, dementia is not prominent in kuru and is indeed characteristically thought to be absent until the late or terminal stage of the disease (Zigas & Gajdusek 1957; Simpson *et al*. 1959; Alpers 1964*b*, 1987; Hornabrook 1968). However, two of the current patients, who were both examined by two senior neurologists, showed definite marked cognitive impairment during stage 2 of the disease. While most formal psychometric tests are linguistically and culturally inappropriate for the Fore, and were therefore not attempted, clinical evidence of multifocal dementia was clearly elicited and has not been previously reported at this relatively early stage in the disease progression, although Alpers (1964*b*) reported disorientation in two patients in the second stage of kuru. In two patients, some sensory changes were reported, with loss of vibration sense in the lower limbs, though joint position sense and pinprick sensation were intact.

The dominant clinical feature of kuru is a progressive cerebellar ataxia. While an ataxic onset is seen in some patients with sporadic CJD, a rapidly progressive cognitive impairment is the central feature. It is of interest that iatrogenic CJD associated with peripheral inoculation with human prions (treatment with contaminated pituitary growth hormone or gonadotrophin) typically presents with ataxia, in sharp contrast to direct CNS exposure by contaminated neurosurgical instruments and dura mater grafting, where the phenotype is generally of classical CJD. This led to suggestions that a cerebellar onset may in part be determined by the route of exposure. Consistent with such an explanation is that in some rodent scrapie models, peripheral exposure is followed by prion replication to high levels in lymphoreticular tissues (Fraser *et al*. 1992*b*), with later neuroinvasion occurring by retrograde spread via autonomic nerves with entry to the spinal cord and then spread to the hindbrain including cerebellum (Beekes *et al*. 1996). vCJD, in contrast to sporadic and iatrogenic CJD, is uniformly associated with high levels of prion colonization of the lymphoreticular tissues (Hill *et al*. 1997*b*, 1999; Wadsworth *et al*. 2001), which has also been detected preclinically in asymptomatic individuals (Hilton *et al*. 1998). This may also reflect route of infection, presumed to be dietary, but could also be determined by prion strain type. As part of our earlier investigation of the diagnostic potential of tonsil biopsy in human prion diseases (Hill *et al*. 1999), a tonsillar biopsy was performed on a single kuru patient. Although a single observation, the lack of any detectable PrP^Sc^ using the highest sensitivity immunodetection methods, during the clinical phase of the infection, argues against there being substantial and uniform lymphoreticular involvement as seen in all cases of vCJD studied, and would be consistent with the prion strain properties of vCJD determining the distinct peripheral pathogenesis of vCJD rather than route of exposure. This observation would suggest that secondary (iatrogenic) vCJD, arising from parenteral rather than oral exposure to vCJD prions, may also be associated with tonsillar infection allowing early diagnosis by tonsil biopsy. This interpretation is supported by positive tonsil analysis in a recent blood transfusion-associated secondary case of vCJD (Wroe *et al*. 2006).

The recognition of the similarities between kuru and CJD (Klatzo *et al*. 1959) and, subsequently, scrapie (Hadlow 1959) led to the experimental transmission of kuru to primates (Gajdusek *et al*. 1966) and, 2 years later, the transmission of CJD (Gibbs *et al*. 1968). This identified the cluster of the transmissible spongiform encephalopathies among diseases of the central nervous system (CNS), which later developed into the unifying concept of the mammalian prion diseases (Prusiner 1982; Collinge 2001). When BSE was first described, it was immediately recognized as a member of this group. There are powerful similarities between BSE and kuru in that they are the only known examples of an epidemic created by the oral intraspecies recycling of infectious CNS material. The oral transmissibility of BSE to humans to cause vCJD provides yet another link between kuru and BSE/vCJD.

In addition to the central historical importance of kuru, the end stage of the kuru epidemic offers a unique opportunity to study the parameters of a nearly completed human prion disease epidemic and its genetic and other determinants. In particular, recognition of the extraordinary incubation periods possible following natural prion infection of humans is important in providing an insight, from actual case histories rather than mathematical models, into the likely span of the vCJD epidemic in the UK. It was not possible to estimate kuru incubation periods early in the epidemic since the actual infecting event for an individual was unknown, though the onset of disease in children as young as 5 years gave a measure of the minimum incubation period—it may have been less than 5 years but it could not have been more. For the maximum incubation period, we now have a new measure since the abrupt and permanent interruption of the source of infection, endocannibalism, in the late 1950s has progressively allowed recognition of an enormous span of possible incubations extending up to, and perhaps beyond, the incubations of more than half a century described here. The significance of these remarkable findings has recently been discussed in detail (Collinge *et al*. 2006).

During our field studies, we have obtained from older people who participated in traditional mortuary feasting explicit accounts of the practices and their social and spiritual significance. These detailed descriptions will be published separately but reaffirmed the oral histories of endocannibalism among the Fore reported by a number of earlier investigators (Glasse 1967; Alpers 1968; Mathews *et al*. 1968; Gajdusek 1977; Lindenbaum 1979). Despite the importance of spiritual cannibalism in religious observance and the use of modern forms of cannibalism in organ and tissue transplantation, the term ‘cannibalism’ and its cognates have an unavoidable derogatory connotation. We therefore prefer to use the term ‘transumption’ (Alpers 1999, 2005) for describing the mortuary practice of consumption by family members of their dead, as practised by the people of the kuru region to incorporate the body of their loved one into the bodies of the living and help to free the newly disembodied spirit. This practice ceased abruptly when the people first came under Australian administrative control. While isolated events may have occurred for a few years after this prohibition, new exposures of individuals to kuru at mortuary feasts would not have occurred after 1960. In fact, no patients with kuru have been recorded who were born after 1959 (and only nine were recorded in those born between 1956 and 1959); indeed, all of the 11 recently recorded cases of kuru, reported here, were born before 1950. Were there any remaining source of infection, whether from surreptitious cannibalism or possible ground contamination (Brown & Gajdusek 1991), we would have expected to have had kuru patients born after this period—particularly, as children are thought to have had shorter incubation periods—but this has not been observed. In addition, no clusters of cases, as shown to have been the outcome of the last family mortuary feasts celebrated in the area (Klitzman *et al*. 1984), have been recorded for many years. We found no evidence for maternal transmission from the kuru archives, from colleagues who had practised medicine in the area or from local oral history. Again, any vertical route of kuru transmission, if it existed, should also have resulted in the occurrence of kuru cases in persons born after 1960, particularly since kuru was common among women of childbearing age; no such cases have occurred. We can therefore confidently attribute the persistence of the epidemic beyond another four decades to long incubation periods.

With respect to extrapolation to incubation periods of BSE prion infection in humans, it is important to recognize that the kuru epidemic arose from human-to-human transmission. The transmission of prions between different mammalian species is associated with a barrier, originally termed a ‘species barrier’ (Pattison 1965) but better called a ‘transmission barrier’ in recognition of the importance of within-species prion strain type in addition to species-specific differences in its determination (Collinge 1999). Such a transmission barrier leads to prolongation of the mean incubation period, an increase in the spread of incubation period and a reduced attack rate in comparison with the 100% mortality generally associated with within-species inoculation with high-titre infectious material. Incubation periods approaching the natural lifespan of the inoculated species are often seen in primary cross-species transmission of prions. The second and subsequent passage of prions within the new species is always associated with considerable shortening of the mean and spread of incubation period and high or total lethality to high-titre inocula. Indeed, transmission barriers are quantified experimentally by measuring the fall in incubation period between primary transmission and subsequent passage in the new host, or by comparison of LD_50_s (the dose causing 50% mortality) between the two species by endpoint titration of infectivity in the two species concerned. For this reason, to estimate the range of possible incubation periods of BSE infection of humans causing vCJD requires superimposition of the effect of a transmission barrier onto the findings of natural incubation periods in human prion disease exemplified by kuru.

The mean incubation period for kuru has been estimated to be approximately 12 years (Collinge 1999) and a similar estimate has been made in iatrogenic CJD associated with the use of human cadaver-derived pituitary growth hormone (Brown *et al*. 2000). As evidenced here, in kuru, maximum incubation periods can exceed 50 years. The cattle BSE-to-human transmission barrier is unknown and cannot be directly determined. However, the cattle-to-mouse barrier for the BSE strain has been well characterized experimentally by comparative endpoint titration. BSE prions transmit readily to laboratory mice (Fraser *et al*. 1992*a*), including following oral dosing (Barlow & Middleton 1990). The murine LD_50_ (assayed in C57Bl/6 mice) is approximately 500-fold higher than that in cattle (Wells *et al*. 1998); this barrier also results in a three- to fourfold increase in mean incubation period (Collinge 1999; Lloyd *et al*. 2002). Mean incubation periods of human BSE infection of 30 years or more should therefore be considered possible, if not probable (Collinge 1999), with the longest incubation periods approaching, and perhaps exceeding, the normal human lifespan. The shortest incubation periods in kuru were of the order of 5 years. Similarly in vCJD, although the total clinical case load to date has been much smaller, the youngest onsets have been at the age of 12 years or more, providing an early estimate of a minimum incubation period.

The importance of prion strain differences, host genetics and their interaction makes prediction difficult across the spectrum of prion diseases. In mice, prion disease follows a well-defined course with a highly distinctive and repeatable incubation time for a given prion strain in a defined inbred mouse line. In addition to the PrP gene, a small number of additional genetic loci with a major effect on incubation period have been mapped (Stephenson *et al*. 2000; Lloyd *et al*. 2001, 2002). It can be anticipated that the human homologues of such loci may play a key role in human susceptibility to prion disease, both following accidental human prion exposure and exposure to the BSE agent. By definition, the patients identified to date with vCJD are those with the shortest incubation periods for BSE. It is possible that these patients received a particularly high dose of BSE prions. However, no unusual history of dietary, occupational or other exposure to BSE has been reported from case–control studies. Given the powerful genetic effects on incubation period in laboratory animals, it is therefore possible that the vCJD patients so far identified represent a distinct genetic subpopulation with unusually short incubation periods to BSE prions, with vCJD occurring predominantly in those individuals with alleles for short incubation time at these multiple genetic loci in addition to having the codon 129 methionine homozygous *PRNP* genotype. It is possible therefore that a human BSE epidemic will be multiphasic, and that recent estimates of the size of the vCJD epidemic based on uniform genetic susceptibility may substantially underestimate the eventual size (Ghani *et al*. 2000; D'Aignaux *et al*. 2001). Genes involved in species-barrier effects, which would further increase both the mean and range of human BSE incubation periods, are also likely to be relevant. In this context, it will be difficult to accurately model a human epidemic until such modifier loci are identified and their gene frequencies in the population can be measured (Lloyd *et al*. 2001).

Heterozygosity at *PRNP* codon 129 is a major determinant of susceptibility to and incubation time of the human prion diseases (Palmer *et al*. 1991; Collinge *et al*. 1991, 1996*a*; Lee *et al*. 2001; Mead *et al*. 2003). Unsurprisingly, most (8/10) of these recent kuru cases, with prolonged incubation periods, were heterozygous. We have previously reported that most elderly survivors of exposure to traditional mortuary feasts are heterozygous (Mead *et al*. 2003). Although the number of patients in our study was necessarily small, there was no evidence of association of kuru with *PRNP* haplotype, HLA *DQ7*, *ApoE* or *PRND* alleles (Collinge *et al*. 2006).

The spread of kuru began from an initial focus in Uwami (figure 2) in the Keiagana, moved to Awande on the boundary of North and South Fore and thence north and south into the Fore. The Fore have had the bulk of the cases, certainly in the period since the investigation of the disease began in 1957. In the peripheral areas, and particularly in those villages in figure 2 with a history of kuru but no recorded cases in the database, kuru was of less significance or died out quickly. The people of the kuru region form overlapping endogamous groups and there is no inherent reason why kuru should have spread in the way it did. This may have happened by chance or owing to differences in the transumption patterns between different clans and linguistic groups or owing to differences in genetic susceptibility. The mortuary practices are being studied in detail and specimens taken for genetic analysis. Although the genetic findings so far, as reported here, show no influence on kuru, with the exception of the effect of codon 129 heterozygosity on incubation period (and possibly clinical duration), it is clear that other genetic loci play a major role in the determination of prion incubation periods in mice (Stephenson *et al*. 2000; Lloyd *et al*. 2001, 2002), and the human homologues of such mouse loci may well also influence kuru incubation periods. Through the current programme of research in the field, questions raised in the early days of kuru research (Gajdusek *et al*. 1961; Glasse 1962; Mathews 1965; Alpers 1968; Mathews *et al*. 1968) may yet find an answer.

## Figures and Tables

**Table 1:** Illness duration, *PRNP* genotype and estimation of incubation period in 11 recent kuru patients (Patients' initials are based on name and coded. M, methionine; V, valine; n.a., not available.).

patient	sex	year of birth	onset	age at onset	death	age at death	duration of illness (months)	*PRNP* genotype	likely^a^ incubation period (years)
YAK	male	1948	Nov 1994	46	Dec 1996	48	25	MV	39
PKW	female	1946	Aug 1995	49	Dec 1996	50	16	MV	
MWK	male	1933	Apr 1996	63	Apr 1997	64	12	MM	56
AKA	male	1949	Nov 1996	47	Jul 1998	49	20	MV	40
AYY	male	1940	Jun 1998	58	Apr 2000	60	22	MV	51
AYA	male	1936	Nov 1998	62	Nov 1999	63	12	n.a.	55
WKW	male	1943	Jan 1999	56	Jun 2000	57	17	MV	49
TAM	female	1945	Mar 1999	54	Jan 2000	55	10	VV	
MAA	female	1944	Apr 1999	55	Jan 2001	57	21	MV	
INO	female	1942	Jan 2000	58	May 2001	59	16	MV	
KAW	male	1943	Oct 2001	58	Apr 2003	60	18	MV	51

a As males are unlikely to have become infected after the age of 6–8 years (see text), a conservative estimate of the likely minimum incubation period can be calculated as the number of years from age 7 to disease onset.
